# Efficacy and safety of lower dose tenofovir disoproxil fumarate and efavirenz versus standard dose in HIV-infected, antiretroviral-naive adults: a multicentre, randomized, noninferiority trial

**DOI:** 10.1080/22221751.2020.1752609

**Published:** 2020-05-04

**Authors:** Jun Chen, Rong Chen, Yinzhong Shen, Hongxia Wei, Xicheng Wang, Renfang Zhang, Zhiliang Hu, Ronghui Xie, Qiong Huang, Jiangrong Wang, Li Liu, Tangkai Qi, Zhenyan Wang, Wei Song, Yang Tang, Jianjun Sun, Hongzhou Lu

**Affiliations:** a Department of Infectious Disease, Shanghai Public Health Clinical Center, Shanghai, People’s Republic of China; b Department of Infectious Disease, The Second Hospital of Nanjing, Nanjing University of Chinese Medicine, Nanjing, People’s Republic of China; c Yunnan AIDS Care Center (YNACC), Yunnan Provincial Infectious Disease Hospital, Kunming, People’s Republic of China

**Keywords:** HIV, tenofovir disoproxil fumarate, efavirenz, low dose, non-inferiority

## Abstract

Reduced doses of antiretroviral (ARV) drugs may lower toxicity while preserving efficacy. We aimed to evaluate the efficacy of reduced doses of both tenofovir disoproxil fumarate (TDF) and efavirenz for the treatment of HIV-1 infection. In this open-label, non-inferiority trial, HIV-1-infected antiretroviral-naive adults were randomly assigned to receive either a lower dose anti-retroviral regimen comprised of TDF (200 mg), efavirenz (400 mg), and standard dose lamivudine (300 mg) or the standard dose regimen. The primary endpoint was the proportion of participants with HIV-1 RNA≤ 50 copies/mL at week 48 using a non-inferiority margin of –10%. At week 48, 79 of 92 (85.9%) participants in the lower dose regimen group and 78 of 92 (84.8%) in the standard dose regimen group achieved HIV-1 RNA≤ 50 copies/mL (treatment difference 1.1%, 95% CI −9.1 to 11.3) in the intention-to-treat analysis. Drug-related adverse events occurred more frequently in the participants receiving the standard dose regimen compared with the lower dose one (63.0% vs 80.4%). Changes in estimated glomerular filtration rate and bone mineral density were comparable between the two groups. The non-inferior efficacy and better safety profile of the lower dose ARV regimen support its use as alternative initial therapy for HIV-1 infected patients.

## Introduction

Despite the availability of new therapeutic drugs, the combination of efavirenz (EFV), lamivudine (3TC) and tenofovir disoproxil fumarate (TDF) is still extensively prescribed worldwide either as separate entities or as a single tablet regimen. Owing to both its efficacy in suppressing HIV replication and relatively low cost, this regimen has been used extensively in the low- and middle-income countries.

However, this combination often causes significant adverse effects, mainly due to EFV. EFV is associated with central nervous system symptoms (e.g. dizziness, headache, insomnia and strange dreams) and rash [1]. More importantly, the use of EFV has been reported to increase the risk for suicidal ideation or attempted or completed suicide [2]. As a result, several studies investigated the possibility of using intermittent, EFV-based regimen as maintenance therapy in virologically controlled HIV-1-infected children, adolescents and adults that demonstrated favourable outcomes [3–6]. More importantly, the landmark ENCORE 1 study, a randomized, double-blind, placebo-controlled, non-inferiority trial reported that a reduced dose of 400 mg EFV was non-inferior to the standard dose of 600 mg at weeks 48 and 96 in antiretroviral-naive adults [7,8]. Therefore, TDF plus 3TC, and a reduced dose of 400 mg EFV was recommended as the alternative first-line regimens by the WHO [9].

In addition to the adverse effects often associated with EFV, TDF is also associated with adverse effects: renal and bone [10]. Renal toxicity can be manifested by decreased glomerular function or proximal tubulopathy which leads to loss of phosphorus and subsequent osteopenia over time [10–12]. Various studies have shown that the reduction in bone mineral density (BMD) in HIV-1 infected patients initiating TDF-containing regimens were approximately 1–3% greater compared to those starting with non-TDF containing regimens after 48 and 144 weeks of ART [13]. To reduce its toxicity, intermittent use or a reduced dose of TDF may be a promising strategy. Pharmacokinetic parameters of TDF are dose proportional: in treatment-experienced patients, TDF provided durable reductions in HIV-1 RNA in a dose-related manner at doses of 75–300 mg daily [14,15]. These results suggest that lower doses of TDF may also be effective even if there is baseline resistance. Indeed, intermittent use of TDF and EFV together either with 3TC or emtricitabine were successful as maintenance therapy in HIV suppressed patients [4,6]. However, evidence for using a lower dose of TDF in treatment naïve patients is still lacking.

The primary objective of this study was, therefore, to evaluate the efficacy and safety of a lower dose regimen of TDF 200 mg, 3TC 300 mg and EFV 400 mg compared with the standard dose regimen in the treatment of HIV-1 infection in ART-naïve adults.

## Methods

### Study design and participants

A randomized, open-label, non-inferiority trial was conducted at three sites (Shanghai, Nanjing and Kunming) in China. Eligible participants at screening (within 28 days before randomization) were HIV-infected adults older than 18 years and willing to initiate ART. Participants were excluded if they were pregnant or were nursing mothers, had uncontrolled active opportunistic or malignant disease, present or recent use of prohibited drugs affecting participation or laboratory values outside predefined ranges (absolute neutrophil count <1500 cells per μL, haemoglobin <9.0 g/dL, platelet count <75,000 cells per μL, creatinine clearance (Cockcroft–Gault equation) at least 90 mL per min, and aspartate aminotransferase, or alanine aminotransferase, or total bilirubin >3 times the upper limit of normal). Potential participants with any demonstrated resistance to TDF, 3TC or EFV at baseline were also excluded.

The study protocol was approved by the Shanghai Public Health Clinical Center Ethic Committee, China, in accordance with the Declaration of Helsinki. Written informed consent was obtained from each participant. The study is registered with ClinicalTrials.gov, number NCT02945163.

### Randomization

We randomly assigned participants (1:1) to receive a standard dose regimen of TDF 300 mg, 3TC 300 mg plus EFV 600 mg or a lower dose regimen of TDF 200 mg, 3TC 300 mg plus EFV 400 mg. Treatment assignment was done in accordance with a central randomization schedule generated by using an online randomization service (www.sealedenvelope.com) [16]. TDF mg tablets were cut and with one in third dropped in Shanghai Public Health Clinical Center as TDF 200 mg tablet was not commercially available in China. TDF were quantified in 20 randomly selected samples to ensure the accuracy of the cutting procedure (see Supplement methods). Blood concentrations of TFV at week 8 were determined in all the participants to further confirm the accuracy of the cutting procedure.

### Study design

Study visits for all participants were planned at baseline and at weeks 4, 8, 12, 24, 36 and 48 and included the following assessments: physical examination, adverse event reporting, biochemistry, haematology, immunology and viral load quantification. Plasma viral load was measured in the clinical laboratory of Shanghai Public Health Clinical Center with the Abbott m2000 Real Time HIV-1 Test (Abbott Molecular, Des Plaines, IL, USA; lower limit of detection 20 copies per mL) while all the other tests were performed at each local site. HIV resistance test was performed if the participant had a plasma viral load higher than that in the previous visits (≥200 copies per mL). Virologic failure was defined as HIV-1 viral load >200 copies per mL load at 48 weeks after starting ART; or HIV-1 viral load ≥200 copies per mL in persons with previously undetectable HIV-VL. The participant was also considered a treatment failure if HIV resistance developed and would be dropped out from the study. Adherence counselling was employed at each visit as per standard of care. Bone mineral density was measured using dual energy X-ray absorptiometry (DXA) at each local site at baseline and week 48.

### Study endpoints

The primary objective of the study was to demonstrate the non-inferior virological efficacy of the lower dose regimen compared with the standard dose regimen, with the primary endpoint being the proportion of participants with plasma HIV-1 RNA <50 copies per mL at week 48. Safety endpoints included the incidence and severity of adverse events according to the WHO toxicity scale.

### Statistical analysis

Based on our real-life data, around 94.9% of the patients received the standard dose regimen had a viral load below 50 copies per mL at 48 weeks. We calculated that one hundred and sixty six participants (83 per group) provided 90% power to exclude a non-inferiority margin of 10% for the difference in proportion of participants reaching the primary endpoint (non-inferiority concluded if the lower boundary of the two-sided 95% CI for the difference in response is greater than −10% between the two groups). To ensure that the per-protocol analysis had 90% power to establish non-inferiority, an additional 18 participants were enrolled accounting a possible lost to follow-up of patients. The intention-to-treat analysis included all randomized participants who received at least one dose of study drug.

Continuous variables were described as mean ± standard deviation or as median and interquartile ranges (IQR) depending upon the distribution. The Student's t test was performed to assess differences between two groups. Wilcoxon rank-sum tests were used for nonnormally distributed data. The association between categorical variables and the different group of treatments was assessed using chi-squared test or Fisher's exact test, where appropriate. Wilcoxon matched pairs signed-ranks test was used to test the equality of matched pairs of observations for bone mineral density and eGFR values at baseline versus week 48 in the different groups of treatments.

The difference in proportion of participants in the two regimens who had HIV RNA <50 copies per mL was estimated and two-sided 95% confidence intervals (CIs) of the difference was calculated using *z*-test for comparison of proportions. The intent to-treat analysis was performed in all the participants who received at least one dose of the regimen. Participants with HIV-1 RNA <50 copies per mL in the week 48 were classified as virological success. All other participants (i.e. those with HIV-1 RNA >50 copies per mL or missing HIV-1 RNA data in the week 48) were deemed as virological failures. Individuals who dropped out for any reason other than virological failure were excluded from the per-protocol analysis. The safety analysis set included all randomly assigned participants who received at least one dose of study drug. All analyses were performed using STATA v12.0 (StataCorp, College Station, TX) and GraphPad Prism 7.0 (GraphPad Software, La Jolla, CA) software.

### Role of the funding source

The funders have no role in the study design, performance, analysis and writing. All authors had access to the analysed data and could assess the results and conclusions.

## Results

**Figure 1.**
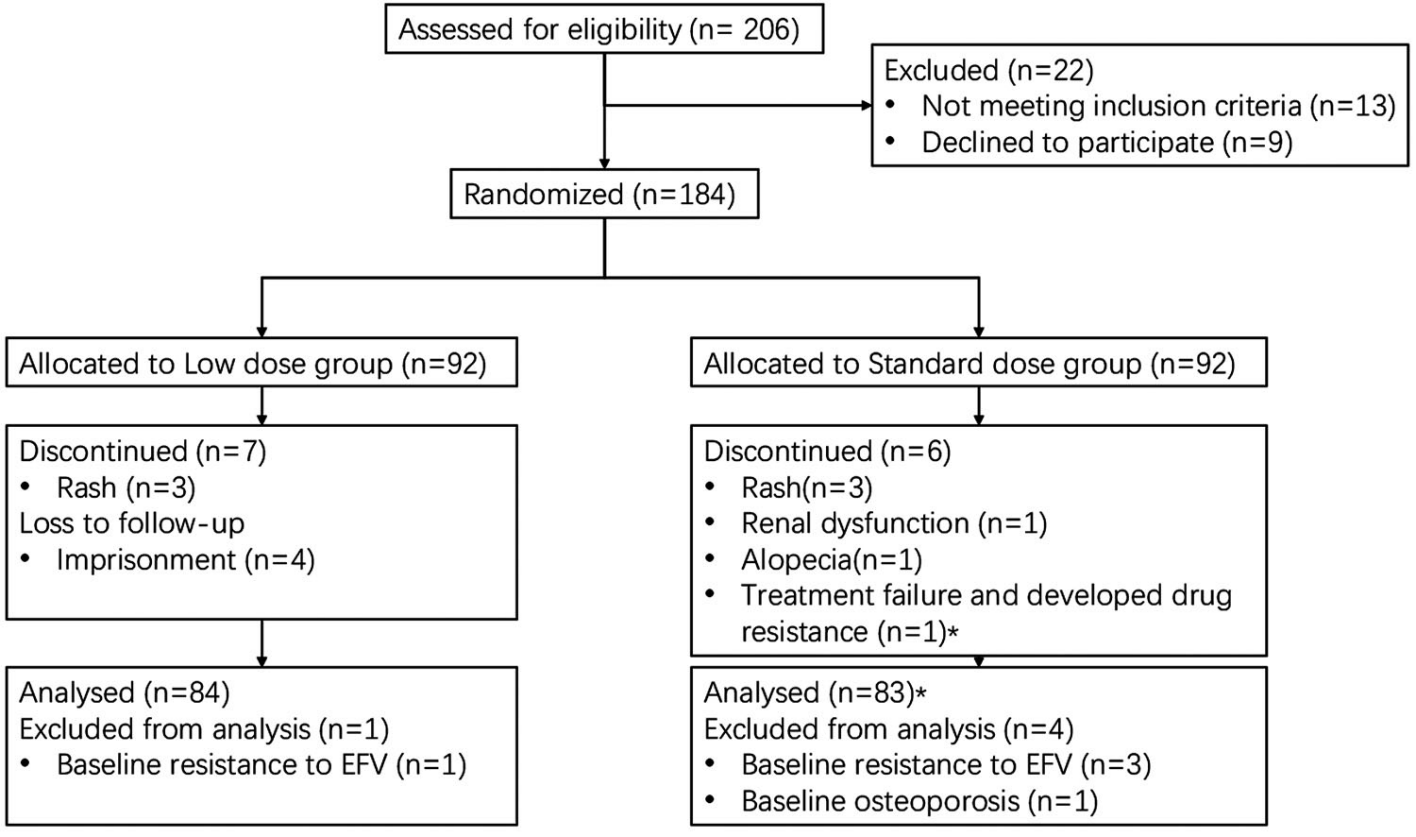
Trial profile. *The patient was analysed in the per-protocol analysis. Three patients were imprisoned for taking drug while one patient because of stolen.

During a 4-month period beginning in March 2018, 206 patients were screened for the study, and 184 were randomized to the two study groups (Figure 1). The last week 48 visit was completed in June 2019. All the participants received at least one dose of the study drug. Baseline characteristic of the 184 enrolled patients were well balanced between the two arms (Table 1). Of note, 170 participants (92.4%) were men with a median age of 30 years (IQR 25–36). The median baseline viral load was 4.5 log copies per mL while median CD4 T-cell count was 292 cells per μL (IQR 199–426). The average BMI was 21.5.

**Table 1.** Demographics and clinical baseline characteristics in the intention-to-treat populations.

	Lower dose group (*n* = 92)	Standard dose group (*n* = 92)	Total (*n* = 184)
Men	83 (90.2%)	87 (94.6%)	170 (92.4%)
Age (years)	31 (25–36.5)	28 (25–36)	30 (25–36)
Weight (kg)	65.0 ± 8.6	63.0 ± 9.3	64.0 ± 8.9
BMI (kg/m^2^)	21.8 ± 2.5	21.2 ± 2.6	21.5 ± 2.6
Median (IQR) plasma HIV RNA in log_10_ copies per mL	4.4 (3.9–4.8)	4.5 (4.2–4.9)	4.5 (4–4.8)
Plasma HIV RNA copies per mL			
≥100,000 copies/mL – no. (%)	14 (15.2%)	13 (14.1%)	27 (14.6%)
Baseline CD4 T-cell count (cells/µL)	310 (190–466)	272 (206–376)	292 (199–426)
CDC stage			
A/B	67 (72.8%)	70 (76.1%)	137 (74.5%)
C	25 (27.1%)	22 (23.9%)	47 (25.5%)
Creatinine clearance (mL/min)	113.9 (105.8–121.8)	112.2 (104.5–120.9)	113.7 (104.8–121.3)

**Figure 2.**
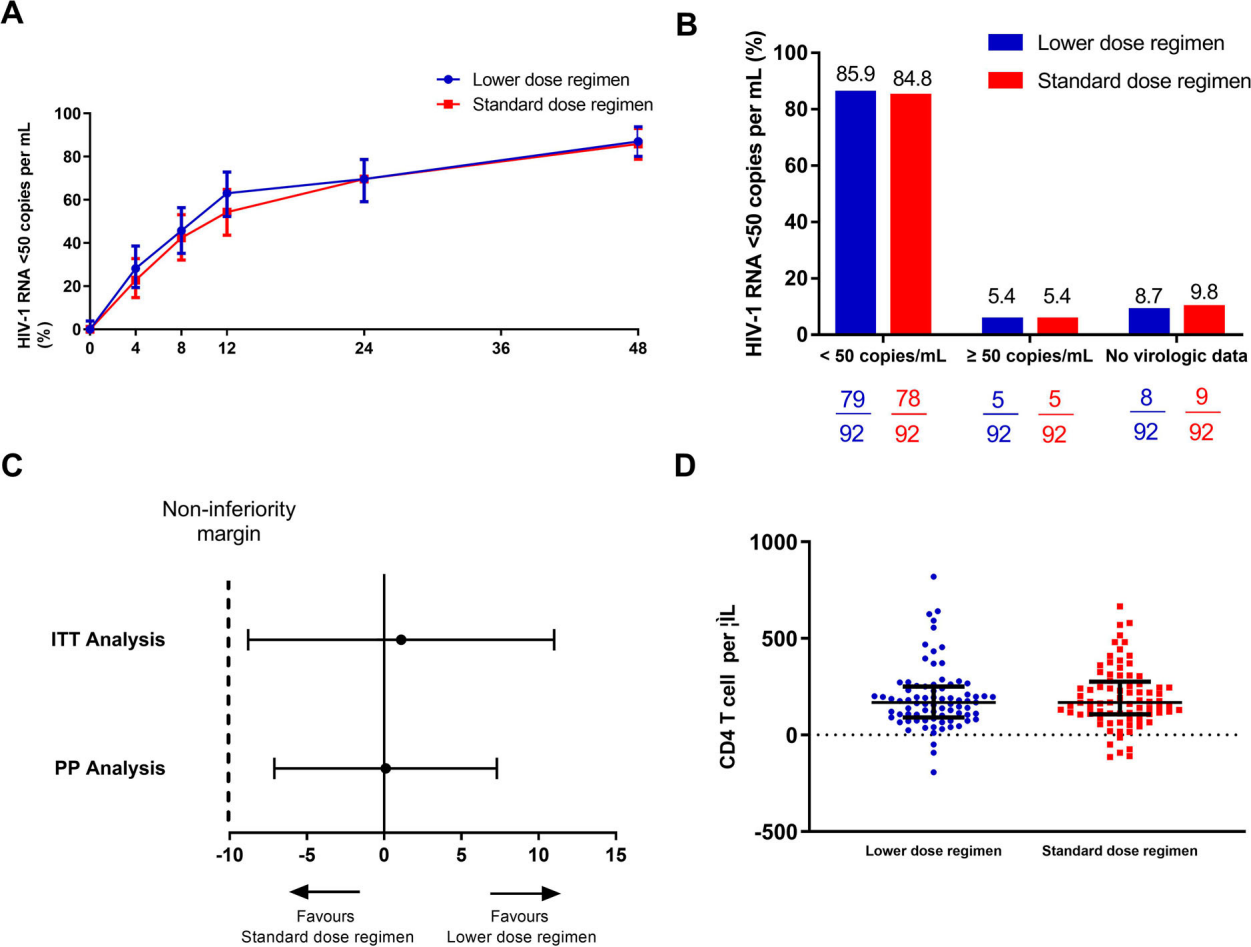
Virological and immunological response of the participants. (A) Snapshot analysis of participants with HIV-1 RNA of less than 50 copies per mL at week 48. (B) Proportion and (C) estimated difference in proportion of participants with HIV-1 RNA of less than 50 copies per mL at week 48, according to intention-to-treat analysis or per-protocol analysis. (D) Immunological response of the participants at week 48. ITT: intention-to-treat analysis; PP: per-protocol analysis.

In the intention-to-treat population, 79 of 92 (85.9%) participants in the lower dose regimen group and 78 of 92 (84.8%) in the standard dose regimen group achieved HIV-1 RNA of less than 50 copies per mL at week 48 according to the FDA snapshot algorithm (treatment difference 1.1%, 95% CI −9.1 to 11.3; Figure 2). The study, therefore, met its primary efficacy endpoint of non-inferior virological efficacy at week 48, as the lower boundary of the two-sided 95% CI for the difference was greater than –10%. The statistical power of this non-inferiority study was 69% based on these efficacy data. In patients with baseline HIV-1 RNA ≥ 100,000 copies per mL, the viral suppression(<50 copies/ml) rate at week 48 was 78.6%(11 in 14) and 76.9%(10 in 13) for the lower dose regimen group and the standard dose regimen group respectively.

A total of 18 participants discontinued treatment: 10 in the standard dose regimen group and 8 in the lower dose regimen group, respectively (Figure 1). The most common reasons for study withdrawal were due to adverse events (8 [44.4%], 3 in the lower dose group and 5 in the standard dose group) while only one patient (1 [5.6%]) withdrew due to lack of efficacy. One patient each in of both groups achieved HIV RNA of less than 50 copies per mL before dropping out due to adverse events. Of the other 16 participants who discontinued treatment, all of them (including one treatment failure patient) achieved HIV RNA of less than 50 copies per mL at week 48 (however, 3 participants in the lower dose regimen group did not have HIV RNA data in the week 48 window).

In the per-protocol population, 79 of 84 (94.0%) participants in the lower dose regimen group and 78 of 83 (94.0%) in the standard dose regimen group achieved HIV-1 RNA of less than 50 copies per mL at week 48 (adjusted treatment difference 0.1%, 95% CI −7.1 to 7.3). One patient (standard dose regimen group) withdraw in week 4 due to high-level resistance to EFV (V106M mutation developed), while another nine patients had HIV-1 RNA of higher than 50 copies per mL at week 48(4 in standard and 5 in lower dose group). Among those who did not reach the end of primary endpoint, the majority of the participants (80% [8/10]) had a low level of plasma viral load (less than 200 HIV RNA copies per mL). 1 in standard and 2 in lower dose group were virological failure. All of them had more than 95% self-reported adherence. HIV genotyping resistance testing demonstrated HIV-associated mutations for 3TC and EFV resistance (M184V, K101E and G190S) in one case in the lower dose regimen group. After 48 weeks of treatment, the increase in CD4 T-cell count from baseline were similar between the two groups (168 [90–250] cells per μL and 167.5 [106.5–275] cells per μL for the lower dose regimen group and the standard dose regimen group, respectively, *P*=0.68, Figure 2D).

During the 48-week study period, there were a total of 345 adverse events (Table 2); 64(69.6%) in the lower dose regimen group reported one or more adverse events while 76(82.6%) reported 1 or more AEs in the standard dose regimen group, respectively. Most of the adverse events were of mild or moderate severity. A significantly lower number of participants in the lower dose regimen reported adverse events (definite or possibly related) to study drug (58 [63.0%] compared with the standard dose regimen 74 [80.4%]; *P* = 0.01; Table 2). However, of these participants, 3 (3.3%) in the lower dose regimen and 5 (5.4%) on the standard dose regimen discontinued study drugs (*P* = 0.72).

**Table 2.** Reported adverse events.

	Lower dose regimen (*n* = 92)	Standard dose regimen (*n* = 92)	Total (*n* = 184)	*P* value
Any adverse events	64 (69.6%)	76 (82.6%)	140 (76.1%)	0.06
Adverse events definitely or probably related to study drug	58 (63.0%)	74 (80.4%)	132 (71.7%)	0.01
Patients stopping drug due to drug-related adverse events	3 (3.3%)	5 (5.4%)	8 (4.3%)	0.72
Serious adverse events	2 (2.2%)	1 (1.1%)	3 (1.6%)	1.0
Serious adverse events definitely or probably related to study drug	0 (0)	1 (1.1%)	1 (0.5%)	1.0
Central nervous system-related adverse events^a^	36 (39.1%)	49 (53.3%)	85 (46.2%)	0.08
Rash	17 (18.5%)	14 (15.2%)	31 (16.8%)	0.69
Gastrointestinal adverse events	4 (4.3%)	12 (13.0%)	16 (8.7%)	0.07

^a^Central nervous system-related adverse events include abnormal dreaming, somnolence, anxiety, cerebellar disorder and ataxia, dizziness, headache and migraine, impaired concentration, insomnia, seizure, depression, fatal suicide, manic reactions and severe depression.

Serious adverse events (SAE) were observed in 3 participants (1.6%) of the study population: 2 (2.2%) in the lower dose regimen group and 1 (1.1%) in standard dose regimen group (*P* = 1.0). Only one serious adverse event (standard dose group, EFV related rash) was judged definitely or probably related to the study drug.

**Figure 3.**
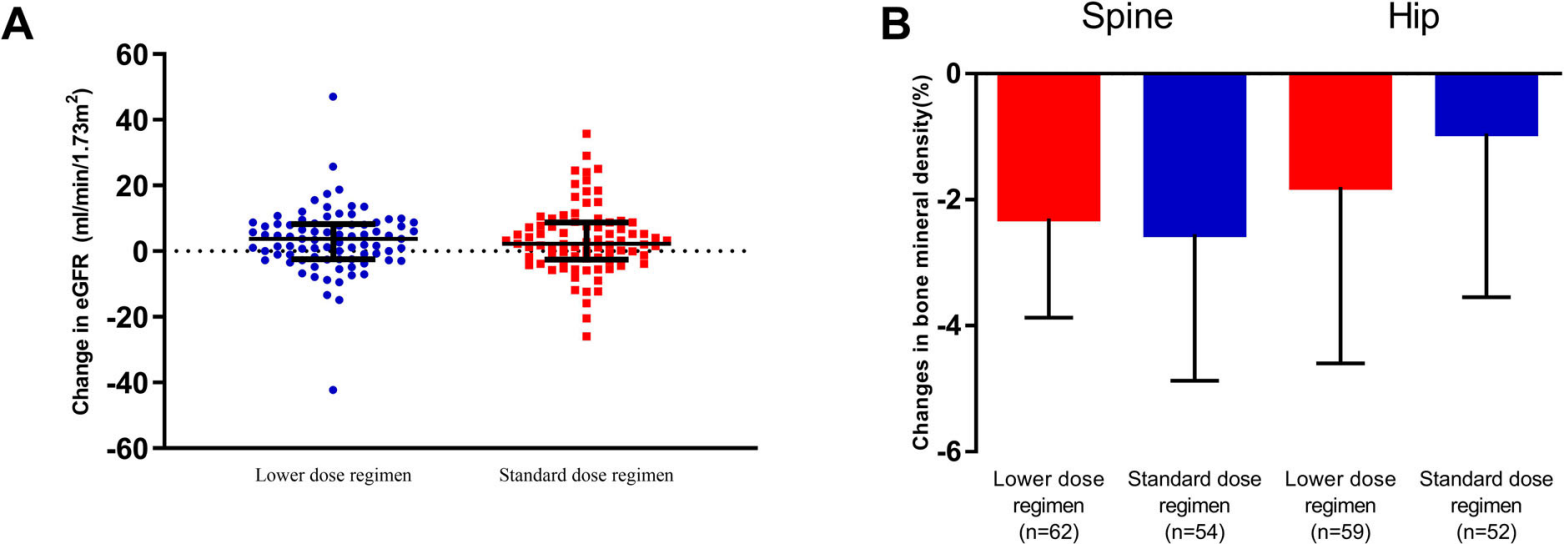
Tenofovir disoproxil fumarate related adverse events. (A) Changes in estimated glomerular filtration rate after 48 weeks’ antiretroviral therapy. (B) Changes in bone mineral density (%) after 48 weeks antiretroviral therapy. Data was shown as median with interquartile range.

The proportion of participants having central nervous system-related adverse events were comparable between the two groups (36 [39.1%] vs. 49 [53.3%], *P* = 0.09). Rash was reported in 17 [18.5%] and 14 [15.2%] of the participants respectively. Elevated liver enzymes occurred in 15 (16.3%) participants in each study group. During the study, the eGFR of 1 patient (1.1%) in the standard dose regimen group decreased sharply from baseline at week 4 and was removed from the study. At 48 weeks, the eGFR increased 3.7 (–2.5 – 8.3) mL/min/1.73 m^2^ in the lower dose regimen group compared with 2.3 (–2.6 – 8.8) ml/min/1.73 m^2^ in the standard dose regimen group (Figure 3A). No significant difference in changes in eGFR between the two groups was found (*P* = 0.85). The BMD of the spine and hip decreased significantly in both of the two study groups at 48 weeks. There were no statistically significant differences in the BMD changes in the spine (–2.3% [–3.9%–0.0%] in the lower dose regimen group or in the standard dose regimen group (2.6% [–4.9% to –0.3%], *P* = 0.67, Figure 3B). Changes in hip BMD were also comparable between the two study groups (–1.8% [–4.6%–1.0%] in the lower dose regimen group vs. –1.0% [–3.6%–1.6%] in the standard dose regimen group, *P* = 0.35).

## Discussion

To our knowledge, this study is the first randomized, controlled trial in an ART-naive population, in which a lower dose regimen, comprised not only of EFV but also of TDF, has been shown to non-inferior to the standard dose regimen of TDF 300 mg plus 3TC 300 mg, and EFV 600 mg. Overall, the frequency of adverse events was comparable between groups and both groups demonstrated a low rate of discontinuations. However, adverse events related to study drugs were significantly more frequent in participants in the standard dose regimen group than in the lower dose regimen.

The efficacy of both the study regimens was satisfactory as only one participant dropped out due to lack of efficacy and around 5 percent of the patients who completed 48 weeks treatment did not achieve HIV-1 RNA less than 50 copies per mL. The virological response rate in the standard dose regimen group was consistent with previous studies that used EFV-containing regimens as the first-line antiretroviral therapy [8,17]. The viral suppression rate in the lower dose regimen group (85.9%) was also similar to that observed in the ENCORE1 trial (82.9%) but higher than that in a recent trial in Cameroon (69.0%) [8,18]. This difference could be explained by the relatively lower proportion of patients with high baseline HIV RNA in the current study (15.2%) compared with that in the ENCORE1 trial (33.3%) and trial in Cameroon (66%).

As mentioned above, we did not find any difference in the overall frequencies of adverse events between the two groups. However, we did observe significantly lower frequency of drug-related adverse events in patients receiving the lower dose regimen as compared with those who received the standard dose regimen. This result was consistent with that in ENCORE1 trial. However, as many as 63.0% of the participants in the lower dose regimen group experienced drug-related adverse events which was significantly higher than that in the ENCORE1 trial (33.8%). This may be due to a relatively low BMI in our study population as compared to that in ENCORE1 trial. Importantly as reported in the Results section above, most of the participants in the current study only had transient symptoms not requiring medical intervention nor were discontinued from the study.

TDF-induced nephrotoxicity has frequently been reported in persons who were co-administrated ritonavir-boosted protease inhibitors. It has also shown to occur in non-PI containing regimens. This AE has been partially explained by increased TDF concentration due to drug–drug interactions and resultant tenofovir accumulation in proximal renal tubular cells [19–23]. As a result, reducing the TDF dose should result in decreased TDF concentrations in renal proximal tubular cells and subsequently lower nephrotoxicity. In the current study, the estimated glomerular filtration rate actually increased slightly in both TDF containing regimens after ART initiation, which has been observed in many clinical trials [8,24–27]. However, no significant difference in eGFR change at week 48 was found between the 2 study groups. This may be due to a relatively young study population with high eGFRs at baseline. As the TDF-induced eGFR decline was time-dependent, longer monitoring of the eGFR changes were also warranted.

BMD decrease usually occurs in the first 48 weeks of ART initiation [28]. In the current study, we found that the BMD of the spine and the hip of both groups decreased significantly at week 48 as compared to baseline and the magnitude of the decrease at these two sites was comparable between the two groups. Our results actually suggest that reducing the dose of TDF did not appear to have effect on BMD during the relatively short 48-week trial.

Reducing the dose of TDF and EFV while preserving virological efficacy could result, at a population level, in cost savings, thus enabling more HIV+ persons to receive treatment. Despite the approval of new antiretroviral drugs (e.g. tenofovir alafenamide [TAF]), TDF is still widely prescribed worldwide. With respect to EFV, its market is shifting to integrase inhibitors. However, EFV-based regimens will likely remain an important part of ART in certain regions and also as an alternative therapy for those who are unable to obtain integrase inhibitors. Consequently, TDF 200 mg plus lamivudine 300 mg, and EFV 400 mg could result in meaningful cost savings even in countries where generic drugs are available, and should, therefore, be considered an important regimen in low and middle incoming countries.

There are some important limitations to the current study. First, although the study was fully powered in the per-protocol analysis, it was underpowered in intention-to-treat analysis as the viral suppression rates were lower according to the FDA snapshot algorithm than we expected. Therefore, a larger, randomized, double-blind, multi-centre clinical trial to verify the current results is still needed. Second, the open-label design is likely to have introduced bias in the interpretation of whether adverse events may have been drug-related. Third, the lack of renal tubular injury markers as well as the exclusion of participants with creatinine clearance <90 mL per min may have limited the potential to have shown a beneficial effect on kidney function of the lower dose. Lastly, the criteria for enrolment into the study did not preclude persons with hepatitis B virus (HBV) infection, but we did not evaluate the anti-HBV efficacy of the lower dose regimen. Therefore, HBV-DNA in patients with HBV infection should be closely monitored if they use this lower dose regimen. And lastly, women were underrepresented in the study. Therefore, the results may not be able to extend to female.

## Conclusion

This randomized, open-label, multi-centre clinical trial demonstrated that a lower dose ARV regimen composed of TDF 200 mg, EFV 400 mg and standard dose lamivudine (300 mg) was non-inferior to the standard dose regimen but with a lower frequency of adverse events.

## Authors’ contributions

JC, YS and HL participated in the conceptualization of the studies. JC, RC and HL were involved with formal data analysis, methodology, project administration and supervision. JC and HL were responsible for funding acquisition. JC, YS, HW, XW, RZ, ZH, RX, QH, LL, JW, TQ, ZW, WS and YT participated in the conduct of the study, including the recruitment and follow-up of participants. JC, RC and HL were responsible for study resources. All authors participated in the drafting and review of the manuscript.
